# Addressing psychosocial vulnerability in rare diseases: a call to action from a European expert consensus study

**DOI:** 10.1186/s13023-025-04017-3

**Published:** 2025-10-27

**Authors:** Rosanne M. Smits, Aukje Aerts, Teodor Angelov, Marissa Bentele, Ivo de Blaauw, Michaela Dellenmark-Blom, Saskia F. A. Duijts, Krister Fjermestad, Monica Franscini, Caterina Grano, Sigrid Hendriks, Laura Inhestern, Thomas Kenny, Charlotte von der Lippe, Loes Oomen, Jan Peter Rake, Andre B. Rietman, Iris A. L. M. van Rooij, Chris M. Verhaak, Nicoline B. M. Voet, Holly Walton, Wendy van Zelst-Stams, Linda Kwakkenbos

**Affiliations:** 1 Department of Medical Psychology, Amalia Children’s Hospital, Radboud University Medical Centre, 9101, 6500 HB Nijmegen, The Netherlands; 2 Department of Child and Adolescent Psychiatry/Psychology, Sophia Childrens Hospital, Erasmus University Medical Centre, Rotterdam, The Netherlands; 3 Department of Neurology, Alexandrovska University Hospital, Medical University of Sofia, Sofia, Bulgaria; 4 Department of Psychosomatic Medicine and Psychotherapy, University Hospital Tübingen, Tübingen, Germany; 5 Department of Pediatric Surgery, Amalia Children’s Hospital, Radboud University Medical Centre, Nijmegen, The Netherlands; 6 Department of Pediatrics, University of Gothenburg Institute of Clinical Sciences, Gothenburg, Sweden; 7 Department of Pediatric Surgery, Sahlgrenska University Hospital Queen Silvia Childrens Hospital, Gothenburg, Sweden; 8 Department of Womens and Childrens Health, Karolinska Institutet, Stockholm, Sweden; 9 Department of Research and Development, Netherlands Comprehensive Cancer Organisation (Integraal Kankercentrum Nederland, IKNL), Utrecht, The Netherlands; 10 Department of Psychology, University of Oslo, Oslo, Norway; 11 Frambu Resource Centre for Rare Diseases, Siggerud, Norway; 12 Department of Psychology, SS Antonio e Biagio e Cesare Arrigo Hospital, Alessandria, Italy; 13 Department of Psychology, Sapienza University of Rome, Rome, Italy; 14 VSOP—Vereniging Samenwerkende Ouder En Patiëntenorganisaties, Soest, The Netherlands; 15 Department of Medical Psychology, University Medical Centre Hamburg-Eppendorf, Hamburg, Germany; 16 Partner Site Hamburg of the German Center for Child and Adolescent Health, Deutsches Zentrum Für Kinder- und Jugendmedizin DZKJ, Hamburg, Germany; 17 Rare Diseases International, Paris, France; 18 Department of Medical Genetics, Telemark Hospital Trust, Skien, Norway; 19 Department of Pediatric Urology, Amalia Children’s Hospital, Radboud University Medical Centre, Nijmegen, The Netherlands; 20 Department of Pediatrics, Amalia Children’s Hospital, Radboud University Medical Centre, Nijmegen, The Netherlands; 21 IQ Health Science Department, Radboud University Medical Center, Nijmegen, The Netherlands; 22 Department of Rehabilitation, Donders Institute for Brain, Cognition and Behaviour, Radboud University Medical Centre, Nijmegen, The Netherlands; 23 Rehabilitation Center Klimmendaal Arnhem, Arnhem, The Netherlands; 24 Institute of Epidemiology and Health Care, University College London, London, UK; 25 Department of Human Genetics, Radboud Institute for Health Science, Radboud University Medical Centre, Nijmegen, The Netherlands; 26 Department of Psychiatry, Radboud University Medical Centre, Nijmegen, The Netherlands; 27 Department of Clinical Psychology, Behavioral Science Institute, Radboud University, Nijmegen, The Netherlands

**Keywords:** Rare diseases, Consensus workshop, Psychosocial factors, Quality of life

## Abstract

**Background:**

Persons living with a rare disease (PLWRD) often encounter burdensome and stressful events that may severely affect their psychosocial vulnerability. There is an urgent need for psychosocial support in PLWRD. We aimed to reach consensus about the most prominent psychosocial needs in rare diseases and future research directions to develop adequate psychosocial support for rare diseases.

**Methods:**

An adapted nominal group technique (NGT) session was conducted in Nijmegen (The Netherlands) with a European expert group of professionals working in the rare disease field. Seventeen participants from seven different European countries took part in the NGT session (seven researchers, two therapists, one patient representative, four clinicians/researchers, and three clinicians), and 23 participants took part in the overall workshop and contributed to this consensus statement.

**Results:**

An initial list of 57 psychosocial needs was aligned with Pickers’ theoretical framework of eight principles of care: patient-centredness, emotional support, access to care, information and education, partner and family involvement, respect and autonomy, care organisation, continuity of care, and physical comfort. For future research directions, six items remained with main focal points addressing international collaborations, inclusivity in rare diseases (patient representatives, ethnic minorities, and elderly), identifying common needs across rare diseases, and translating psychosocial models from common chronic conditions.

**Conclusions:**

A consensus meeting was organised that primarily addressed the psychosocial vulnerability in rare diseases. With the outcomes of this study, we aim for better representation of psychosocial vulnerability within health care and to create future research directions to reduce psychosocial vulnerability in rare diseases.

**Supplementary Information:**

The online version contains supplementary material available at 10.1186/s13023-025-04017-3.

## Background

Rare diseases are characterized by a wide diversity of symptoms and signs that vary not only from disease to disease but also from patient to patient suffering from the same disease [1]. In this manuscript, the term"rare disease"will be employed, in accordance with the terminology established by the European Patient Network (EURORDIS) and the European Reference Networks (ERNs). While"rare disease"will be the primary term used, it is acknowledged that terms such as"rare disorder"or"rare condition"are also encompassed within this classification and may be understood as equivalent in this context. There are up to 8000 known rare diseases, and despite the large variation in rare diseases (e.g. rare vs. ultra-rate, congenital or diagnosed later in life, visible vs. less visible), there are common burdensome and stressful events related to the rarity that is different from individuals with a more common disease [2–4]. Psychosocial support is needed for persons living with a rare disease (PLWRD). For example, PLWRD experience a significant delay in the diagnostic process of approximately five years [5, 6]. Other adverse experiences are heterogeneity of symptoms, limited options for (curative) treatment, scarcity of information, complex care coordination, miscommunication with health care professionals, and difficulty finding the right practical, financial, and psychosocial support [4, 7–9]. These experiences lead to reduced mental well-being and psychosocial vulnerability [10]. For example, a EURORDIS global survey study (Rare Barometer) found that 37% of people affected by a rare disease suffer from depression symptoms, which is more than three times higher than that of the general population [10]. Furthermore, research indicates that mothers of children with rare diseases experience higher levels of anxiety and depression symptoms compared to mothers of children with more common chronic illnesses. This supports the idea that the rarity of the disease presents unique challenges that go beyond those faced by parents of children with other illnesses [11–13]. Moreover, health-related quality of life has also been reported to be lower in rare diseases than in more common conditions [3]. Unfortunately, due to the large number of knowledge gaps in rare diseases, psychosocial topics or health-related quality of life have often been outside research agendas. It has only been since recent years that psychosocial vulnerability in rare diseases has gained more attention [7, 3, 14, 4].

Currently, there is an urgent call from international and national patient organisations to promote mental well-being and reduce psychosocial vulnerability in rare diseases and complex conditions [7, 10, 11, 15]. However, intervention studies within the psychosocial area on how to adequately support PLWRD is lacking, and psychosocial vulnerability studies in rare diseases are scarce. To address the urgency for the representation of psychosocial vulnerability within healthcare and research, a two-day workshop was organised in Nijmegen (the Netherlands) that united European clinicians, therapists, patient representatives, and researchers to discuss topics that focus on psychosocial vulnerability, psychosocial impact, and health-related quality of life in rare diseases. At the end of the workshop, a brainstorming session took place with the aims of (1) Specifying the most prominent psychosocial needs in rare diseases across European countries and (2) Discussing future research direction to develop adequate support that takes psychosocial vulnerability in rare diseases into account. The consensus method Nominal Group Technique (NGT) was used to reach a consensus on both topics [16].

## Method

A two-day Rare Together Workshop was held on May 8 and 9th, 2023, in Nijmegen, the Netherlands. The Rare Together Workshop was organised to form a platform to advocate for psychosocial vulnerability in rare diseases and promote knowledge exchange amongst European researchers, clinicians, therapists, and patient representatives. Participants were recruited based on publications and involvement in psychology and rare diseases and via snowballing in adjacent research networks. The two-day workshop comprised presentations on psychosocial vulnerability and psychosocial topics in rare diseases, networking activities, and closed with a 2 h brainstorm adapted NGT session. In total, 23 participants attended the workshop, and 17 participants participated in the NGT session (7 researchers, 2 therapists, 1 patient representative, 4 clinicians/researchers, and 3 clinicians; see Table 1 for an overview of the participants). To include all participants for a complete consensus statement, the results of the NGT were later distributed to all participants for revision and additional contributions.

**Table 1:** Participants of the rare together workshop (N = 23)

Profession (n, %)	Country (n)
Researcher (10, 43.5%)	Bulgaria (1), The Netherlands (5), Italy (1), United Kingdom (1), Germany (1), Norway (1)
Clinician^a^/Research (6, 26.1%)	Sweden (1), The Netherlands (3), United
Clinician^a^ (4, 17.4%)	Kingdom (1), Norway (1)
Therapist (2, 8.7%)	The Netherlands (3), Italy (1)
Patient Representative (1, 4.3%)	Germany (1), The Netherlands (1) The Netherlands (1)

^**a**^Clinicians included a pediatric surgeon, general practitioner, rehabilitation physician, psychologists, and clinical geneticist

### Nominal group technique

**Fig. 1:**
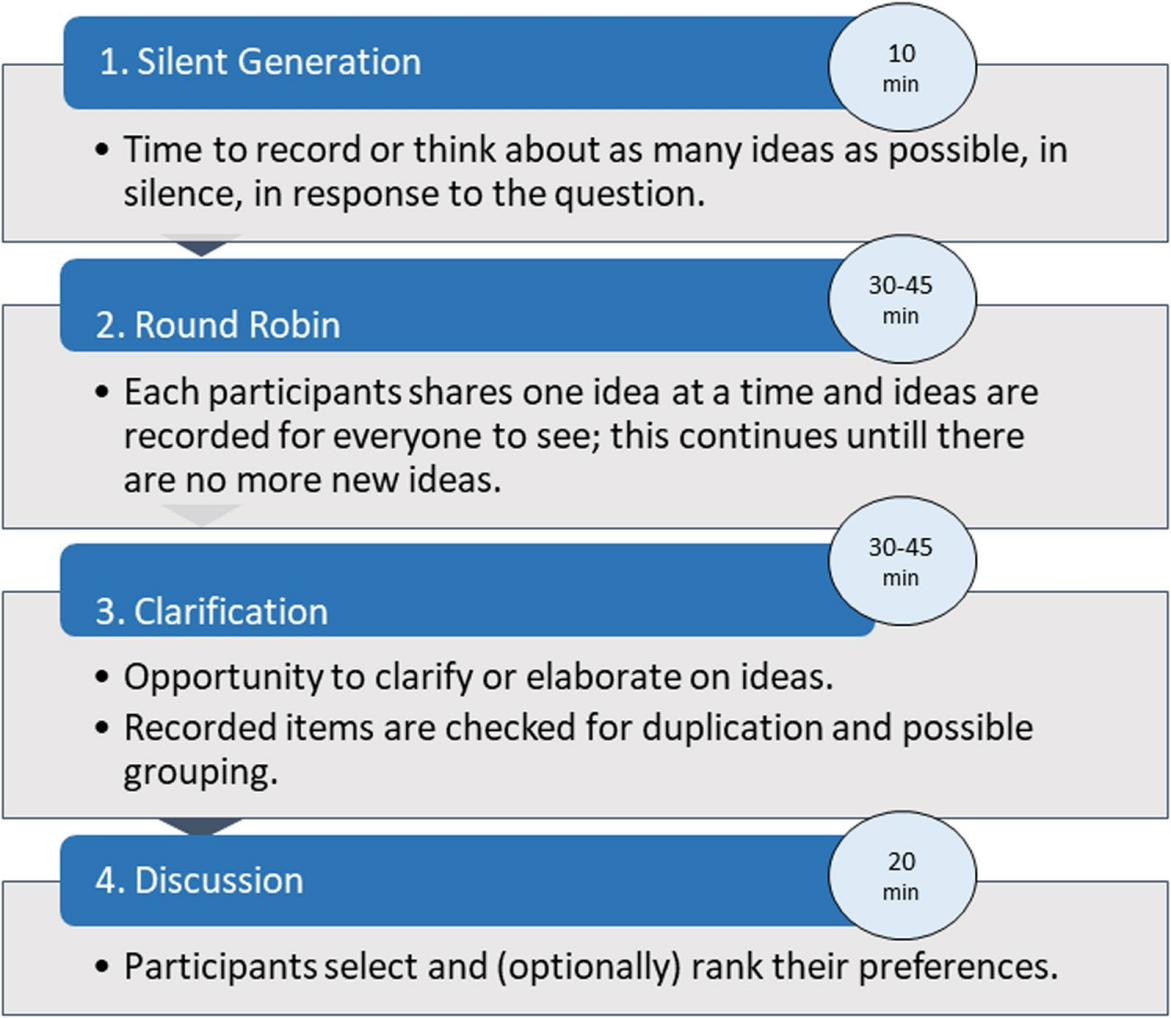
A model of the NGT process with time stamps from the current NGT session [17, 19]

NGT is a structured face-to-face group interaction approach that effectively enhances participant empowerment by allowing them to voice their opinions and have their viewpoints considered by fellow group members [16, 17]. Delbecq and Van de Ven originally devised the NGT, which comprises four essential stages: silent generation, round robin, clarification, and discussion (and/or ranking) (Fig. 1) [16–18]. The current NGT session focused on two core research questions, to: (1) Specify the most prominent psychosocial needs in rare diseases across European countries and (2) Discuss future research direction to develop adequate support for psychosocial vulnerability in rare diseases. Because the NGT session took place at the end of a two-day workshop with presentations that discussed topics related to mental well-being and psychosocial needs, there was no need to include a general introduction. During the silent generation (10 min), participants took time to silently generate ideas or solutions to a specific problem, writing them down individually. Subsequently, each participant took turns sharing their ideas (round robin), where the facilitator recorded the ideas on a board. This is done without any discussion or judgment of the ideas, ensuring all contributions are heard. This step ensures that everyone can contribute without influence from others, typically done within a set time limit. After round robin, ideas were discussed during ‘clarification’. In this step, the group clarified and discussed the meaning of each idea to ensure everyone understood them. This step focuses on explaining and elaborating on ideas, but not evaluating or debating them. Lastly, during the discussion participants vote on the ideas individually, typically ranking or selecting their top choices. The results are tallied, and the most popular or highly-ranked ideas are prioritized for further discussion or action [16–18]. The current NGT session was adapted in that the clarification and voting processes were combined to deduce the psychosocial needs into categories to reach a consensus on the most important ones (‘ranking’). All steps from the NGT session are depicted in Fig. 1.

### Data analysis

After the session closed, three authors (RMS, LK, and CMV) subsequently categorized the statements. To ensure the inclusion of all relevant care aspects, the authors utilized the ‘Picker’ framework for person centred care, as proposed by [20]. This framework encompasses eight essential care dimensions: emotional support, access to care, information and education, partner and family involvement, respect and autonomy, care organisation, continuity of care, and physical comfort [21]. One investigator (RMS) initially read all responses and categorized statements. Then, that investigator and LK and CMV together reviewed the categorized list and discussed any potential discrepancies in categorizing approaches. Agreement on groupings and any revisions were based on consensus. (Table 2).

**Table 2:** List of statements categorized in dimensions of patient-centeredness

1. Dimensions of patient-centerednes^a^	Number of items
Emotional support	20
Access to care	5
Information and education	11
Partner and family involvement	5
Respect and autonomy	8
Care organisation	7
Continuity of care	2
Physical comfort	4
Total (without duplicates)	57

^a^According to the picker institute’s model of patient-centred care

## Results

The nominal group technique session generated an initial list of 62 statements after several rounds of the round robin phase. As a first step during the clarification phase, 57 statements were assigned to the first aim: (1) Specifying the most prominent psychosocial needs in rare diseases, and six items were assigned to the second aim, (2) Addressing future research direction to develop adequate support for psychosocial vulnerability in rare diseases. For the first aim, the Pickers’ eight principles of patient-centredness were used as a framework to categorize all items. In five cases, the items fitted into two categories and were therefore categorized in both (See supplementary file for the list of items). For the second aim, the remaining list of items was discussed, focusing on future research directions and moving forward from the current needs assessment (Table 3).

**Table 3:** Future research directions

2. Focal points for future directions	Number of items
Research for general and specific psychosocial topics in rare diseases	3
Translating psychological models from	1
common conditions	1
Representation	1
International collaborations	1
Total (without duplicates)	6

### Pickers’ eight principles of patient-centredness

#### Emotional support

When focusing on the emotional support of the individual with a rare disease, several recurring needs came forward. The need for psychological support during the diagnostic process, dealing with uncertainty, living grief, and support to remain in daily life were frequently mentioned. For example, participants discussed that during the diagnostic process, there is often no psychological guidance, even though this is a very stressful and potentially traumatic time for patients and their families. Another example is the need to develop coping skills to deal with uncertainty. Participants discussed that uncertainty is omnipresent in rare diseases, which can cause high levels of stress and anxiety. For example, uncertainty can be experienced during the diagnostic process, in relation the future, in the prognosis of the condition, and what it means for daily life. Because uncertainty is a prominent phenomenon in caring for individuals with rare disease patients, psychosocial vulnerability should be closely monitored or aided with adequate coping styles.

#### Access to care

Respondents mentioned that care should be made more accessible across all countries, not only in Western countries. Moreover, individuals with rare diseases address that they often feel unheard by medical staff or have trouble navigating the health care system. Participants also vocalized that rare disease care needs to be more accessible across professions and lifespans and they expressed the need for greater access to hybrid care. Health literacy and bureaucracy were mentioned as two barriers that prevent access to care.

#### Information and communication

Several unmet needs were addressed concerning education and information provision for people with rare diseases and health care professionals. The most frequently discussed topics were the lack of available medical information, reliable information, and knowledge on quality of life and the psychosocial impact of rare diseases. Another frequently addressed topic was raising awareness of rare diseases in medical and psychological education.

#### Partner and family involvement

Family involvement is a critical component of patient-centred care that affects the quality of care and patient outcomes. Besides the family, other systems need to be included in care and play an important role in a patient's life, such as networks like school or work. Several important needs were addressed to facilitate the inclusion of these important systems. For example, there should be more focus on empowerment of the family system, including siblings (e.g., with sibling interventions), and care should involve the entire network surrounding the child, such as the involvement of teachers, siblings, and peers (e.g., with developing teacher brochures that include information about disease management).

#### Respect and autonomy

Several items underlined that PLWRD need to be involved in more studies and policymaking. Another point that patients often address is that medical information, for example, about genetic up make, can be so complex that it is hard to involve patients in shared decision-making. Moreover, respondents acknowledged that a common concern from people with rare diseases is that they feel unheard by healthcare professionals. Another concern is that there is almost no representation or ambassadorship in rare diseases.

#### Care organisation

All participants agreed that to promote and implement psychosocial advances for individuals with a rare disease, several healthcare changes need to occur to facilitate these advances. For example, efforts should be made to motivate young clinicians to specialize in rare disease and to define specific competencies for rare disease health professionals. Moreover, participants emphasized that more attention should be paid to patients with clinical symptoms without a diagnosis because this group is often overlooked.

#### Continuity and transition of care

Transition to adulthood, help with navigating the health system, and navigating bureaucracy were topics that were mentioned as important factors for continuity of care. For example, an often voiced concerns was the ability to plan and coordinate care because of bureaucratic barriers or unclear pathways from paediatric to adult care.

#### Physical comfort

Pain management, striving for normalcy, and assistance with activities and daily living needs were mentioned as ways to support individuals'physical comfort. Leisure time and opportunities for quality time or ‘disease-free’ time were also mentioned as important facilitators of physical comfort.

### Future research to develop adequate support for psychosocial vulnerability in rare diseases

#### Research for general and specific psychosocial topics in rare diseases

All participants agreed it is important to research the overarching challenges in common diseases to develop more general rare disease care needs. Identifying these common challenges, for example, by approaching care from a family system and biopsychosocial approach, will also put forth topics that overlap with more common conditions. On the other hand, specific topics particularly prominent in rare diseases need to be recognized. Conversely, this creates two broad research lines: (1) What are the overarching aspects of rare diseases that are also recognizable in more common severe conditions, and (2) What are the specific challenges of living with a rare condition as such?

#### Translating evidence-based models from common conditions to rare diseases

A second focal point for future research is to investigate which evidence-based interventions or theoretical models from behavioural medicine in more common conditions (e.g., asthma or diabetic care) can be applied and translated to rare diseases. All participants recognized the value of existing interventions (e.g., cognitive behavioural therapy or mindfulness-based interventions) and encouraged their application in rare diseases.

#### International collaboration

As a third focal point to realize the proposed future research directions, more international partnerships and collaborations should be established, for example to stimulate funding and include more diversity in research and patient groups, and to obtain adequate sample sizes.

#### Representation

Finally, there should be a more inclusive representation of rare diseases. Several groups are understudied, such as ethnic minorities and older adults. Moreover, all participants recognized and agreed that more patient representatives should be invited to the upcoming meeting to further define unmet needs and develop measurement tools that can help aid these needs.

## Discussion

This study brought together rare disease researchers, clinicians, and a patient representative to primarily address psychosocial vulnerability in rare diseases. A nominal group technique session was conducted in Nijmegen (The Netherlands) with a European expert group of professionals working in the rare disease research field to (1) specify the most prominent psychosocial needs in rare diseases and (2) discuss future research direction to develop adequate support for psychosocial vulnerability in rare diseases. A list of 57 psychosocial need items was identified, and six recommendations were given for future research.

Generally, most items discussed psychosocial needs and the urgency of addressing and supporting psychosocial vulnerability in rare diseases. This shift in discussion towards psychosocial awareness was not surprising because there is emerging evidence about psychosocial needs in rare diseases, and several studies have pointed out the severity of these needs [4, 9, 14, 22–25]. These studies, for example, mention difficulties in health care navigation, psychological support during the diagnostic process, the impact on family and sibling well-being, impaired quality of life, and the negative experiences recognized by patients of various rare disease (Bogart & Irvin, 2017; [4, 22, 26–30]. Many of the authors of those studies were also present during this consensus meeting. While previous research has highlighted these issues, this meeting offered a structured and collaborative format to define priorities in psychosocial care. This structured initiative marks an important step in consolidating expertise on psychosocial vulnerability. For the categorization of psychosocial needs in rare diseases, we utilized Picker’s eight principles of patient-centeredness as a framework. The results of our study revealed a range of needs that aligned with the aspects of patient-centered care, with emotional support emerging as the category that encompassed the most items. The outcomes of the current study are in line with previous studies, for example with a qualitative study from our research group where the need for emotional support encompassed the most items [29]. Moreover, a recent meta-analysis also uncovered that family members may experience a need for psychosocial support, as they report increased psychological distress [11].

The discussion on future research recommendations was less extensive because most participants recognized the need for research and underlined the importance of investigating overarching psychological concepts specific to rare diseases that already developed models from common conditions cannot capture. Furthermore, participants encouraged the translation of general psychosocial themes, such as adaptation to disease or family-system interventions, as these general approaches are also relevant for rare diseases. There are some examples of comprehensive clinical guidelines for specific rare disorders, e.g. for sex chromosome disorders [31, 32]. Such guidelines are the result of comprehensive work over time that requires expertise not only in the rare disorder but also from multiple medical domains. Hence, the feasibility of such work depends on extensive multi-disciplinary international collaboration. Altogether, it is evident from the discussion that there is a recognized gap between the existing research on the psychosocial impact of rare diseases and the development of concrete plans or policies to address the identified needs. The 2030 Recommendations, particularly the fourth goal of reducing psychological, social, and economic vulnerability by one-third, serve as a benchmark for progress [10]. The current initiative to organise this meeting with prominent authors on psychosocial vulnerability to address the psychosocial impact of rare diseases, aligns with the objectives of the 2030 Recommendations. Despite increasing recognition of these needs, sustained investment and structural support remain limited. As noted in the RARE2030 knowledge base, many initiatives in this area have been discontinued or lack funding, highlighting the importance of long-term commitment to psychosocial care [33].

The reflections on this consensus meeting provide valuable insights for improving future initiatives. A key limitation was the limited representation of individuals with lived experience because there was only one patient representative, namely from the VSOP (Dutch Patient Alliance for Rare and Genetic Diseases). For future research meetings we encourage to include more patients, family members and patient representatives to capture their valuable input in developing a needs assessment. The analytic process was based on independent coding followed by consensus discussions among researchers, which enhanced consistency, although inter-rater reliability was not formally assessed. Incorporating mixed-method designs that compare patient and professional perspectives, and using qualitative analysis tools, may strengthen future studies. Another limitation was the overrepresentation of participants from specific regions, particularly the North and Western parts of Europe. Regional differences can influence the psychosocial needs and challenges faced by individuals with rare diseases and their families. Conversely, many discussed psychosocial needs are also recognized in papers from other countries such as the US, Australia, or Central/Eastern Europe [3, 34, 35]. However, it would also be insightful to gain more knowledge about which aspects individuals in other regions of the world consider important.

## Call to action

In recent years, there has been a growing recognition of the significant psychological vulnerability experienced by individuals with rare conditions [3, 5, 6, 9, 10, 15, 23, 25, 29, 30, 36]. While the field of behavioural medicine has made substantial progress in addressing the psychological aspects of more common diseases, this progress has not been mirrored in the context of rare diseases [29]. The unique challenges faced by individuals with rare conditions, including limited access to specialized care and the psychological burden of uncertainty, remain largely underexplored within the broader landscape of behavioural medicine.

Currently, 94% of rare diseases lack an approved medical treatment [37]. In moving forward, PLWRD need to be psychosocially supported due to this lack of medical treatment. Therefore, it is necessary to advocate for integrating psychosocial care into broader healthcare policies and frameworks, fostering ongoing support and attention to the unique needs of individuals and families affected by rare diseases. Additionally, exploring avenues for sustained international collaboration and securing long-term funding commitments can contribute to developing and implementing effective strategies to reduce psychosocial vulnerability in this population. On a more positive note, in the last years new initiatives are arising such as the Mental Health & Wellbeing Partnership Network from EURORDIS (launched in 2023) that will soon publish a Mental Health Toolkit on their website.

In conclusion, this study contributes to a growing body of work highlighting the need for psychosocial support in rare diseases. Its outcomes offer direction for future research and underline the importance of integrating psychosocial vulnerability into rare disease care and policy.

## Supplementary Information

Additional file1.

## Data Availability

The dataset was built based upon the items generated by the consensus meeting. Data (item list) is available and included in Supplementary file.

## Abbreviations

PLWRD: Persons living with a rare disease
NGT: Nominal group technique
ERN: European Reference Network
