# Supplementary material for: Addressing psychosocial vulnerability in rare diseases: a call to action from a European expert consensus study
Source: Orphanet J Rare Dis. 2025 Oct 27;20:543. doi: 10.1186/s13023-025-04017-3 (PMC12560521; doi:10.1186/s13023-025-04017-3)
Supplement: Supplementary file 1 — Additional file1. [file 13023_2025_4017_MOESM1_ESM.docx]

**Supplementary File**. Rare Together consensus meeting

| 1. **Dimensions of patient-centerednes^s^** | **Number of items** |
| --- | --- |
| Emotional support | 20/21 |
| Access to care | 5/5 |
| Information and education | 11/13 |
| Partner and family involvement | 5/5 |
| Respect and autonomy | 8/11 |
| Care organization | 7/7 |
| Continuity of care | 2/2 |
| Physical comfort | 4/4 |
| Total (without duplicates) | 57 |
| *^a^According to the Picker Institute’s model of patient-centred care* | |

**List of statements**

*=statements categorized in two different categories

**Emotional support (e.g. alleviation of fear and anxiety)**

1. Proactivity in identifying psychosocial needs (emotional support)
2. Determine resilience and vulnerabilities (emotional support)
3. Dealing with uncertainty (emotional support)
4. Psychological support during diagnosis (emotional support)
5. Fear of the future (emotional support)
6. Dealing with stigma, visible and invisible problems (emotional support)
7. Sexuality (emotional support)
8. Fertility/Will i have children/inheritance (emotional support)
9. Techniques for living well with uncertainty (emotional support)
10. All delay during diagnostic process: during diagnostic process / at gp/ anticipatory anxiety for diagnosis (emotional support)
11. How to identify psychosocial needs/high risk group (emotional support)
12. Investigate what works/coping strategies/striving for normalcy*
13. Interventions important developmental stages / social roles (emotional support)
14. Subjects from positive psych/increasing coping and resilience/ACT (emotional support) (respect and autonomy)
15. A place to talk about emotions and needs/safe space (emotional support)
16. Severe mental illness (e.g., psychosis) (emotional support)
17. Treatment burden(emotional support)
18. Living bereavement (emotional support)
19. Coping with trauma from childhood / holistic care / interdisciplinary team (emotional support)
20. Early risk / awareness of involvement psychology/social issues/ adequate timing (emotional support)

**Access to care (e.g. transportation or scheduling appointments)**

1. Access to psychotherapy (psych comorbidity)
2. Accessible experts across professions (Access to care)
3. Access to hybrid care (Access to care)
4. Health literacy (access to care)

**Information and education**

1. Education of teachers, employers etc (information and education)
2. Ask patients who needs to know what when* (information and education) (respect and autonomy)
3. Feeling heard, communication by healthcare professionals (information and education) (respect and autonomy)
4. Awareness for RD in society (information and education)
5. Informational needs (information and education)
6. Inheritance/genetics (information and education)
7. Clear communication about treatment options(information and education)
8. Finding reliable information/checklist (information and education)
9. Information (what information for the child) / tailoring to whom/when/how(information and education)
10. Tools to make difficult decisions(information and education)
11. Information about qol after diagnosis(information and education)

**Partner and family involvement**

1. Belonging to groups (esp. those without peer group) (partner or family involvement)
2. Social support through network (e.g. teachers) (partner and family involvement)
3. Empowerment of family system incl siblings (partner and family involvement)
4. Involve complete network surrounding the child / school/neighbours (partner and family involvement)
5. Schoolsystem: more support in navigation(partner and family involvement)

**Respect and autonomy (respect for patients’ values, preferences and expressed needs)**

1. Ask patients who needs to know what when* (information and education) (respect and autonomy)
2. Subjects from positive psych/increasing coping and resilience/ACT* (emotional support) (respect and autonomy)
3. Feeling heard, communication by healthcare professionals* (information and education) (respect and autonomy)
4. Specification for patients without a group: undiagnosed (respect and autonomy)
5. Meaning in society/ social roles and participation (respect and autonomy)
6. Inequality (respect and autonomy)
7. More awareness in society of having a RD / ambassadorship (respect and autonomy)
8. Understudied groups/ethnic minorities/older adults (respect and autonomy)

**Care organization (coordination and integration of care**

1. Multidisciplinary/involve or raise awareness for all HCP (care coordination)
2. Define/identify specific competencies for RD healthcare professionals (coordination of care)
3. Motivate young clinicians to work for RD (coordination of care)
4. Multidisciplinary/involve or raise awareness for all HCP (coordination of care)
5. Including RD in medical communication (for every involved profession) including taboos (coordination)
6. More international studies/cross country studies (care coordination)

**Continuity and transition of care**

1. Transition to adulthood (continuity of care)
2. Help navigating health system (coordination of care)
3. Navigating bureaucracy (access to healthcare)

**Physical comfort (e.g.** **pain management and assistance with activities and daily living needs)**

1. Opportunities for quality time/disease free time / leisure time (physical comfort)
2. Investigate what works/coping strategies/striving for normalcy (physical comfort) (emotional support)
3. Remaining daily life / support in this (physical comfort)
4. More generic symptoms: pain and fatigue itch (physical comfort)

| 1. **Focal points for future directions** | **Number of items** |
| --- | --- |
| International collaborations | 1 |
| Inclusivity | 1 |
| Identifying overarching needs | 3 |
| Translating psy models from common conditions | 1 |
| Total (without duplicates) | 6 |
|  | |

**Research directions**

1. Identifying common challenges across rare diseases
2. Coping strategies for uncertainty* (emotional support)
3. Apply insights from common disorders
4. Understudied groups/ethnic minorities/older adults* (respect and autonomy)
5. Define ‘unmet needs, develop unmet needs measurement tools (from rare cancers to RD
6. More international studies/cross country studies* (care coordination)
